# Chronic Upregulation of Cleaved-Caspase-3 Associated with Chronic Myelin Pathology and Microvascular Reorganization in the Thalamus after Traumatic Brain Injury in Rats

**DOI:** 10.3390/ijms19103151

**Published:** 2018-10-13

**Authors:** Andriy O. Glushakov, Olena Y. Glushakova, Tetyana Y. Korol, Sandra A. Acosta, Cesar V. Borlongan, Alex B. Valadka, Ronald L. Hayes, Alexander V. Glushakov

**Affiliations:** 1 Department of Neurosurgery, University of South Florida College of Medicine, Tampa, FL 33612, USA; aoglushakov@gmail.com (A.O.G.); sacosta@health.usf.edu (S.A.A.); cborlong@health.usf.edu (C.V.B.); 2 Department of Neurosurgery, Virginia Commonwealth University, Richmond, VA 23298-0631, USA; avaladka@gmail.com (A.B.V.); rhayes@banyanbio.com (R.L.H.); 3 Department of Psychology, Uppsala University, 75142 Uppsala, Sweden; koroltanya@gmail.com; 4 Banyan Biomarkers, Inc., Alachua, FL 32615, USA; 5 Single Breath, Inc., Midlothian, VA 23113-1074, USA

**Keywords:** chronic TBI, cleaved-caspase-3, thalamus, blood-brain barrier, microvascular reorganization, myelin pathology

## Abstract

Traumatic brain injury (TBI) is associated with long-term disabilities and devastating chronic neurological complications including problems with cognition, motor function, sensory processing, as well as behavioral deficits and mental health problems such as anxiety, depression, personality change and social unsuitability. Clinical data suggest that disruption of the thalamo-cortical system including anatomical and metabolic changes in the thalamus following TBI might be responsible for some chronic neurological deficits following brain trauma. Detailed mechanisms of these pathological processes are not completely understood. The goal of this study was to evaluate changes in the thalamus following TBI focusing on cleaved-caspase-3, a specific effector of caspase pathway activation and myelin and microvascular pathologies using immuno- and histochemistry at different time points from 24 h to 3 months after controlled cortical impact (CCI) in adult Sprague-Dawley rats. Significant increases in cleaved-caspase-3 immunoreactivity in the thalamus were observed starting one month and persisting for at least three months following experimental TBI. Further, the study demonstrated an association of cleaved-caspase-3 with the demyelination of neuronal processes and tissue degeneration in the gray matter in the thalamus, as reflected in alterations of myelinated fiber integrity (luxol fast blue) and decreases in myelin basic protein (MBP) immunoreactivity. The immunofluorescent counterstaining of cleaved-caspase-3 with endothelial barrier antigen (EBA), a marker of blood-brain barrier, revealed limited direct and indirect associations of cleaved caspase-3 with blood-brain barrier damage. These results demonstrate for the first time a significant chronic upregulation of cleaved-caspase-3 in selected thalamic regions associated with cortical regions directly affected by CCI injury. Further, our study is also the first to report that significant upregulation of cleaved-caspase-3 in selected ipsilateral thalamic regions is associated with microvascular reorganization reflected in the significant increases in the number of microvessels with blood-brain barrier alterations detected by EBA staining. These findings provide new insights into potential mechanisms of TBI cell death involving chronic activation of caspase-3 associated with disrupted cortico-thalamic and thalamo-cortical connectivity. Moreover, this study offers the initial evidence that this upregulation of activated caspase-3, delayed degeneration of myelinated nerve fibers and microvascular reorganization with impaired blood-brain barrier integrity in the thalamus might represent reciprocal pathological processes affecting neuronal networks and brain function at the chronic stages of TBI.

## 1. Introduction

Traumatic brain injury (TBI) is a major public health problem affecting over 1.7 million Americans annually and causing 52,000 deaths and over 90,000 long-term disabilities [1,2,3,4]. Disabilities from TBI may include problems with cognition, motor function and sensory processing as well as behavioral deficits and mental health problems such as anxiety, depression, personality change and social unsuitability. Posttraumatic headaches (PTH) are among the most common complications, occurring with a prevalence rate of 47–95% in moderate TBI and about 20–38% in moderate-severe TBI patients [5]. Chronic sleep disturbances are also common after TBI, affecting about 50% of patients [6,7]. Furthermore, TBI is associated with chronic neurological and behavioral deficits, including neurological and neurodegenerative diseases such as chronic traumatic encephalopathy, posttraumatic epilepsy, Alzheimer’s and Parkinson’s diseases [8,9,10,11,12,13,14,15,16].

A wealth of clinical data has documented that TBI causes significant brain atrophy, which is also associated with progressive neuronal degeneration and degeneration of the gray and white matter. This may disrupt brain connectivity within days after injury and continue for years [17,18,19]. Anatomical and functional changes in the thalamus and thalamo-cortical circuits may underlie acute and chronic neurological pathologies associated with functions within the thalamo-cortical system [20,21,22,23,24,25,26]. In addition, a significant impairment in cerebral blood flow in the thalamic regions of patients with mild TBI as compared to healthy controls [27].

Anatomical and functional changes in the thalamo-cortical network are associated with neurological outcomes in many chronic comorbid conditions and complications associated with TBI. These conditions include tinnitus, neurogenic pain, major depression syndrome and Parkinson’s disease [8,9,28]. The detailed molecular and cellular mechanisms involved in the etiopathology of these chronic disorders following TBI are not completely understood.

Experimental and clinical data further indicate that many chronic neurological disorders, including TBI and neurodegeneration, share many common features, such as inflammatory and neurovascular pathologies associated with blood-brain barrier extravasation, abnormal angiogenesis and apoptosis [29,30,31]. Activation of caspase-3 is a well-recognized hallmark of neuronal apoptosis in many central nervous system (CNS) disorders and this pathway has long been considered a promising therapeutic target [32,33]. It is widely recognized that apoptosis associated with caspase-3 upregulation in neuronal and glial cells contributes to TBI pathology [34,35,36]. Numerous clinical studies have demonstrated upregulation and activation of caspase-3 and other apoptotic and proteolytic markers associated with its activity in the human brain, cerebrospinal fluid (CSF) and blood samples from patients with acute brain injury, including TBI (see review by Glushakova and colleagues [37]). Our recently published preclinical data demonstrated unrecognized pathologies of chronic TBI in the white matter of the corpus callosum, such as delayed punctate blood-brain barrier opening and the occurrence of microbleeds similar to those often observed in neurodegenerative disorders. Importantly, these pathologies were associated with neuroinflammation, white matter damage and myelin loss affecting brain connectivity [38]. Our data further suggested that caspase-3 activation might represent key mechanisms involved in development of these pathologies [39].

Here, we expand our previous studies to investigate possible myelin pathologies after experimental TBI and the role of apoptosis in the thalamus, a key brain structure involved in the functioning of mammalian cortical networks [40]. We hypothesized that the primary cortical injury causes progressive white matter damage, resulting in disruption of brain connectivity particularly affecting the thalamo-cortical network, further triggering neurodegenerative processes in susceptible thalamic regions. Delayed apoptosis may represent a previously unrecognized chronic pathway mediating delayed and evolving neurodegeneration following brain trauma and contributing to chronic neurological complications and neurodegenerative diseases. To test this hypothesis, we determined spatiotemporal profiles of changes in a well-recognized marker of apoptosis, cleaved-caspase-3 and its association with myelin and blood-brain barrier pathologies using immunohistochemistry in a preclinical model of TBI. 

## 2. Results

### 2.1. Upregulation of Cleaved-Caspase-3 Expression in the Thalamus Following CCI

**Figure 1:**
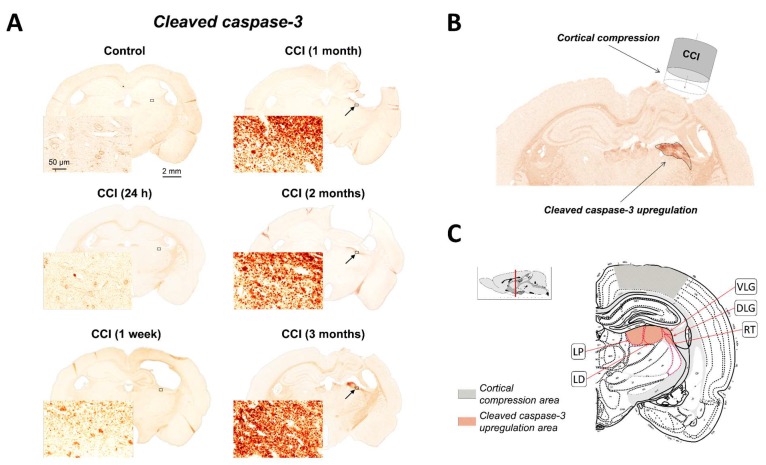

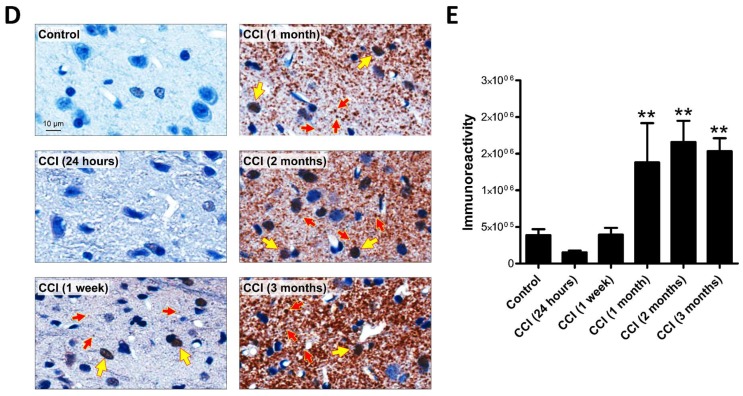
Chronic upregulation of cleaved caspase-3 in the thalamus after experimental TBI. (**A**), Representative photomicrographs of brain sections immunostained with cleaved-caspase-3 in control (sham) and CCI-injured rats at different time points after experimental TBI. Color deconvolution of images (ImageScope, Aperio Technologies, Vista, CA, USA) was used for detection of cleaved-caspase-3 immunoreactivity visualized with DAB stain (brown). Zoomed excerpts shown at the bottom of each brain section illustrate evident upregulation of cleaved-caspase-3 in selected thalamic areas circumscribed by rectangles. Arrows indicate thalamic regions with the most prominent upregulation of cleaved-caspase-3 at time points between 1 and 3 months after CCI. (**B**), Scaled schematic representation of the injury site showing area of a representative brain section at 3 months after CCI highlighting gray matter in thalamic region with the highest level of cleaved-caspase-3 immunoreactivity used in the quantitative analyses. (**C**), Schematic representation of the specific regions affected by caspase-3-mediated apoptosis in the thalamus. The image from a reference atlas indicates the stereotaxic location of coronal brain sections used in analyses (see sagittal view of the brain in excerpt). Thalamic regions affected or partially affected by caspase-3-mediated apoptosis indicated by cleaved-caspase-3 upregulation are shown in call-outs (LD, lateral dorsal nucleus of thalamus; DLG, dorsal part of the lateral geniculate complex; VLG, ventral part of the lateral geniculate complex; LP, lateral posterior nucleus of thalamus; RT, reticular nucleus of thalamus). Area filled with a red crosshatched pattern indicates thalamic area with the most prominent cleaved-caspase-3 upregulation. Area filled with a gray crosshatched pattern indicates cortical regions affected by CCI. (**D**), Representative images of the selected regions of the thalamus corresponding to locations shown in panel A (black rectangles). Morphological features of cleaved-caspase-3 immunoreactivity in control and CCI-injured rats are shown at different time points after experimental TBI. Cleaved-caspase-3 immunoreactivity was visualized with DAB stain (brown) and cell nuclei were visualized with hematoxylin (blue). Cleaved-caspase-3 immunoreactivity was present as punctate staining (brown dots) predominantly with extracellular and to a lesser extent with cellular localization suggested by hematoxylin counterstain (see examples of cleaved-caspase-3-positive cells indicated with yellow arrows). Sparse extracellular cleaved-caspase-3-immunopositive puncta were observed at one week after CCI (see examples of single cleaved-caspase-3 puncta indicated by red arrows). However, although the presence of single puncta is apparent at all time points starting at one week, the most abundant appearance of the cleaved-caspase-3-immunopositive puncta with increased staining intensity and, possibly, evolving aggregation was observed between one and three months after experimental TBI. (**E**), Quantitative analyses of cleaved-caspase-3 immunostaining in the thalamus from control and CCI-injured rats at different time points after experimental TBI. ** *p* < 0.01, one way ANOVA, CCI vs. control (*n* = 5–9 rats per group).

Following experimental TBI, increases in cleaved-caspase-3 immunoreactivity were observed in selected brain regions with the most prominent upregulation detected in selected ipsilateral thalamic regions at sub-acute and chronic time points (Figure 1A). Figure 1B,C also show a scaled schematic rendering of the location of primary CCI injury. The increased expression of cleaved-caspase-3 was localized within the ipsilateral hemisphere and no or only marginal cleaved-caspase-3 expression was observed within the contralateral hemisphere. Interestingly, the highest chronic upregulation of cleaved-caspase-3 following experimental TBI was observed in the thalamus, which is not directly impacted by CCI. In contrast, no prominent chronic increases in cleaved-caspase-3 immunoreactivity were observed in close vicinity to the injury site or in the cortical areas surrounding the injury location. No detectable increases in cleaved-caspase-3 immunoreactivity were observed in control animals at any time point after sham injury. Notably, maximal increases in cleaved-caspase-3 immunoreactivity were observed in dorsal thalamic areas proximate to the hippocampal formation including the lateral posterior thalamic nucleus (LP), the laterodorsal thalamic nucleus (LD), the dorsal lateral geniculate nucleus (DLG) and the ventral lateral geniculate nucleus (VLG) (Figure 1B,C). Immunoreactivity also partially extending into other surrounding areas such as the reticular thalamic nucleus (RT), the intergeniculate leaf (IGL) and the intramedullary thalamic area (IMA) was observed. No detectable increases in cleaved-caspase-3 immunoreactivity were observed in the medial and ventral thalamic regions such as the mediodorsal thalamic nucleus (MD), the posterior thalamic nuclear group (Po), the ventral posterolateral thalamic nucleus (VPL), the ventral posteromedial thalamic nucleus (VPM), the centrolateral thalamic nucleus (CL) and the oval paracentral thalamic nucleus (OPC).

Cleaved-caspase-3 accumulation in the thalamus at subacute time points was predominantly detected as punctate staining, either singular or forming aggregates, with surrounding cleaved-caspase-3 diffuse immunoreactivity showing increasing density at more extended time points (Figure 1D). In addition, counterstaining with hematoxylin revealed the presence of cleaved-caspase-3-immunopositive cells. A very limited appearance of cleaved-caspase-3-immunopositive structures in selected thalamic regions was observed at subacute time points starting one week after CCI, whereas the most abundant chronic accumulation of cleaved-caspase-3 immunoreactivity was observed from one to three months after experimental TBI. Although sporadic appearance of some cleaved-caspase-3-immunoposive puncta was detectible one week after CCI (see excerpt in Figure 1D), its amount was insufficient to produce statistically significant changes in total immunoreactivity at this time point. Large accumulations of cleaved-caspase-3-immunoposive puncta were observed between 1 and 3 months after injury. These morphological observations were consistent with quantitative analyses revealing statistically significant increases in cleaved-caspase-3 immunoreactivity in the thalamus during this time period (Figure 1E). The immunoreactivity values measured at chronic time points from 1 through 3 months were also significantly different compared to those measured at acute and sub-acute time points at 24 h and 1 week after CCI, respectively, with significance levels between *p* < 0.001 and *p* < 0.05.

### 2.2. Neuronal Demyelination and Brain Tissue Degeneration in the Gray Matter of the Thalamus Are Associated with Cleaved-Caspase-3 Upregulation Following CCI

**Figure 2:**
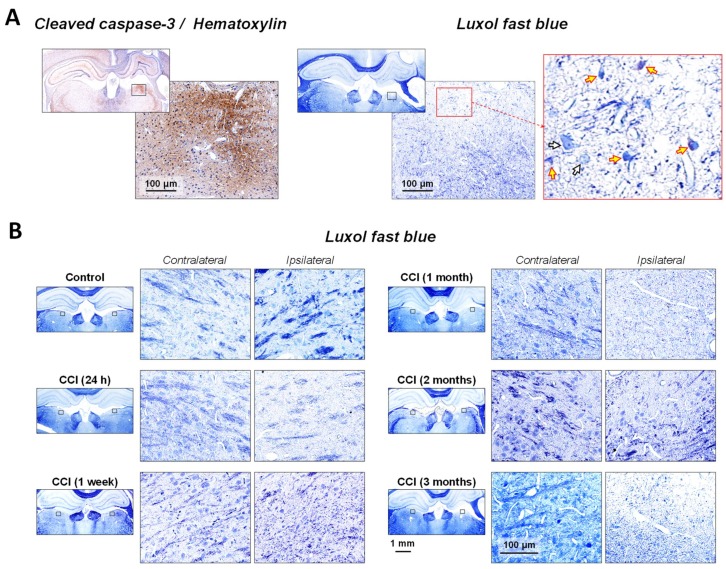
Association of cleaved-caspase-3 upregulation with chronic myelin pathology in the thalamus after experimental TBI. (**A**), Representative photomicrographs of adjacent brain sections immunostained with cleaved-caspase-3 and luxol fast blue counterstained with cresyl violet in the brains of CCI-injured rats at 3 months after experimental injury. Zoomed excerpts of the thalamus (LD) demonstrate the locations of maximal cleaved-caspase-3 immunoreactivity and corresponding maximal decrease in myelin density and cell loss. High magnification excerpt outlined with red (zoom 5×) demonstrates altered brain tissue myelin integrity (luxol fast blue) and cellular morphology (cresyl violet) in the area corresponding to maximal increase in cleaved-caspase-3 upregulation. The white-filled arrows indicate examples of viable cells with distinctive neuronal morphology (i.e., light blue stained cytoplasm with visible nuclei). The yellow-filled arrows indicate examples of impaired cells at different stages of dying with altered morphology (i.e., irregular shaped cells with dark blue stained cytoplasm). (**B**), Representative photomicrographs of luxol fast blue/cresyl violet staining of brain sections of CCI-injured rats at 24 h, 1 week, 1 month, 2 months and 3 months after experimental injury and corresponding control. Zoomed excerpts demonstrate progressive decrease in myelin density and loss of integrity of myelinated fibers (luxol fast blue) as well as changes in cellular morphology (cresyl violet) in the LD of thalamus, one of the thalamic regions associated with the most profound upregulation of cleaved caspase-3, suggesting possible demyelination or loss of myelinated fibers in the ipsilateral thalamus. Rectangles in the low-power images denote location of the zoomed excerpts in ipsi- and contralateral hemispheres.

Our published data suggested that upregulation of cleaved-caspase-3 might be associated with white matter lesions in the corpus callosum starting at day 1 and progressing at least to 3 months after experimental TBI [38,39]. In order to analyze the integrity of myelinated neuronal fibers in the gray matter of thalamic regions affected by experimental TBI, we used counterstaining employing luxol fast blue and cresyl violet to visualize myelin degradation and neuronal degeneration in thalamic regions associated with cleaved-caspase-3 upregulation. The greatest levels of demyelination following experimental TBI were observed in the parts of the thalamus with the most prominent cleaved-caspase-3 upregulation. Figure 2A shows two adjacent brain sections immunostained with cleaved-caspase-3 or stained with luxol fast blue and counterstained with hematoxylin and cresyl violet. The zoomed excerpts in this figure indicate that the areas with the maximal increases in cleaved-caspase-3 immunoreactivity were also associated with maximal levels of demyelination. These areas were also characterized by profound cell loss. Progressive thalamic demyelination was detected as early as 24 h, with the greatest loss occurring at 3 months after CCI as compared to the myelin integrity of corresponding contralateral regions (Figure 2B). In addition to the overall loss of myelin, decreases in numbers of cresyl violet-stained cells in the ipsilateral portion compared to the contralateral counterparts were observed at acute time points, suggesting progressive cell death in thalamic regions following injury. Interestingly, at one week following CCI, there was a marked increase in cresyl violet-stained material presented as scattered, intensively stained, smaller sized and distorted cellular structures in ipsilateral thalamic regions compared to the morphological features of corresponding contralateral regions. The cytomorphological features of these cresyl violet-stained cells reflected microglial characteristics and were distinctive from the cytomorphological features of neurons detected by Nissl staining [41]. Based on our previous findings and published studies, this observation is consistent with the presence of proliferating microglial cells in the injured thalamic regions at sub-acute time points [42,43]. No obvious restoration of the myelin strands in the injured part of the gray matter in the thalamus was evident at later chronic time points as compared to contralateral counterparts. 

**Figure 3:**
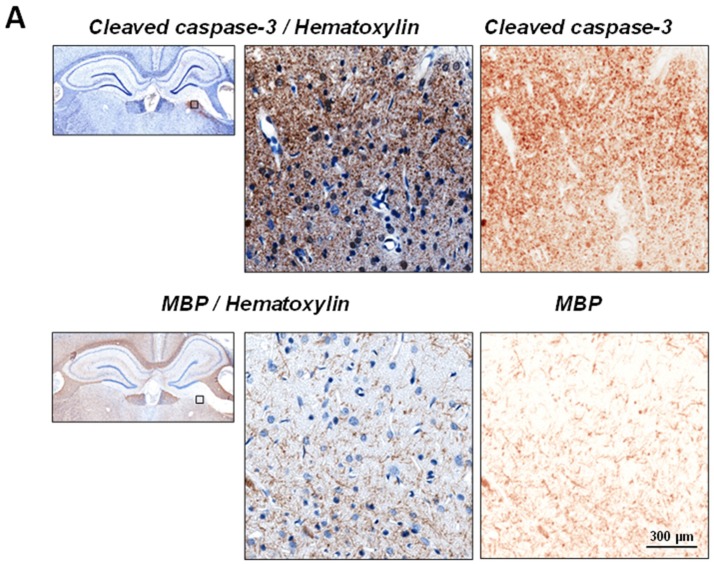

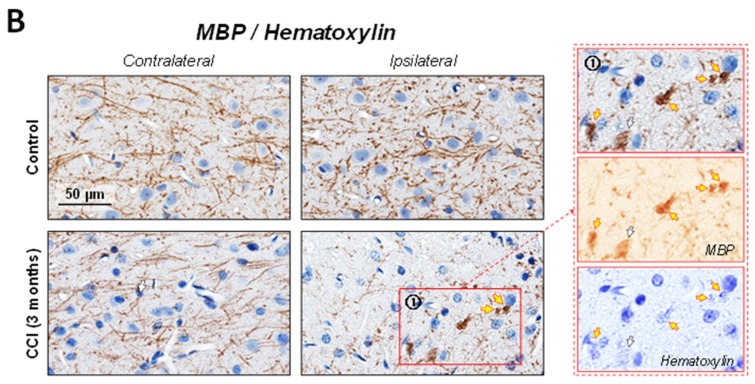
Association of cleaved-caspase-3 upregulation with changes in MBP expression in the thalamus after experimental TBI. (**A**), Representative photomicrographs of adjacent brain sections immunostained for cleaved-caspase-3 and with myelin basic protein (MBP) in the brains of CCI-injured rats at 3 months after experimental injury. Both immunostains were counterstained with hematoxylin to visualize nuclei. Zoomed excerpts of the thalamus (LD) demonstrate the locations of maximal cleaved-caspase-3 immunoreactivity and corresponding maximal decrease in MBP-immunoreactivity. Right panels show corresponding color deconvolution images to demonstrate DAB visualization of changes in immunoreactivity to cleaved-caspase-3 (dark brown stain demonstrates an increase) and MBP (lack of staining or light brown stain demonstrates a decrease). These images demonstrate that, although apparent loss of integrity of MPB-positive structures and decreases in staining intensity were observed in the area characterized by highest density and intensity of cleaved-caspase-3-positive puncta, the overall alterations in MBP-immunoreactivity are only partially associated with cleaved-caspase-3 upregulation. (**B**), Representative photomicrographs of ipsi- and contralateral thalamic sections (LD) immunostained with MBP in the brains of CCI-injured rat at 3 months after experimental injury and corresponding control. Immunostains were visualized with DAB (brown) and the sections were counterstained with hematoxylin (blue) to visualize nuclei. The images demonstrate loss of integrity MBP-positive fibers in the ipsilateral thalamic regions compared to their contralateral counterpart and corresponding thalamic regions of control animal. The arrows indicate MBP-positive aggregates associated with dying cells (yellow-filled arrows) or possibly located extracellularly (white-filled arrows). Excerpts show zoomed area of the section shown with rectangle (1) and color deconvolution images demonstrating specific DAB visualization of MBP-positive structures (brown) and hematoxylin visualization of the nuclei (blue).

To further investigate myelin pathology in the thalamus, immunohistochemical analyses of MBP-immunostained sections were performed in the thalamic regions corresponding to increased upregulation of the cleaved caspase-3. These experiments revealed evident loss of integrity of MBP-positive fibers in the affected ipsilateral thalamic regions as compared to the same regions in the contralateral hemisphere at chronic time points after CCI but not in control animals. Figure 3A demonstrates two adjacent brain sections immunostained with cleaved-caspase-3 and MBP and counterstained with hematoxylin at 3 months after CCI. The zoomed excerpts indicate areas with maximal increases in cleaved-caspase-3 immunoreactivity and decreases in MBP immunoreactivity, suggesting that cleaved-caspase-3 upregulation might be associated with loss of MBP-immunopositive fibers. However, the areas characterized by increased immunoreactivity to cleaved-caspase-3 were more widespread than those characterized by decreases immunoreactivity to MBP. The quantitative analyses revealed that decreases in overall MBP-immunoreactivity in the ipsilateral thalamic areas corresponding to increased cleaved-caspase-3 upregulation compared to its contralateral counterpart were observed only at 3 months after CCI to 55.47 ± 13.30% of the contralateral side value (*n* = 4 rats per group), these changes were not statistically significant. Although the data obtained in these experiments were overall consistent with the luxol fast blue staining and showed decreased expression of MBP in affected ipsilateral thalamic regions compared to contralateral counterparts at 3 months after CCI, the changes in MBP-immunoreactivity were observed to a lesser extent.

MBP-immunostaining experiments also revealed morphological alterations in selected ipsilateral thalamic areas associated with cleaved caspase-3 upregulation. Figure 3B shows representative photomicrographs of MBP-immunostained brain sections counterstained with hematoxylin in control and CCI-injured rats at 3 months after experimental TBI. These changes include loss of MBP-immunopositive fibers and the presence of MBP-immunopositive aggregates. Morphological analyses suggest that the MBP-immunopositive aggregates are co-localized with distorted matter stained with hematoxylin, suggesting that these MBP aggregates are associated with cellular structures at varying degrees of dying which can, possibly, eventually remain in brain tissue as extracellular formations (Figure 3B). Additionally, regions with marked decreases in MBP immunoreactivity were also characterized by reductions in the number of viable cells as suggested by hematoxylin counterstaining. The most profound decreases in MBP-immunoreactivity and tissue integrity were observed in the superior areas of the thalamus, notably LD, located proximate to the hippocampus, which are characterized by myelin loss evident from the luxol fast blue stating experiments (Figure 2 and Figure 3).

### 2.3. Association of Microvascular Reorganization and Loss of Blood-Brain Barrier Function in the Thalamus with Cleaved-Caspase-3 Upregulation after CCI

**Figure 4:**
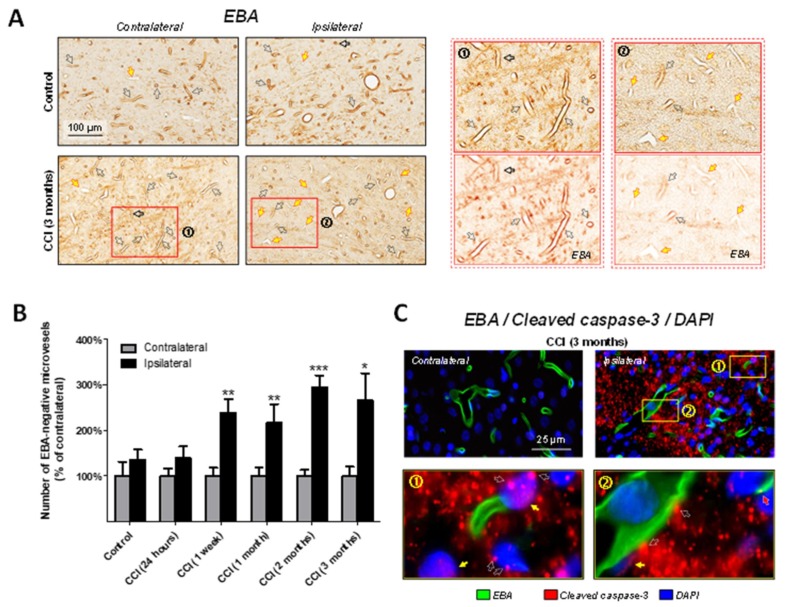
Association of the cleaved-caspase-3 upregulation with blood-brain barrier reorganization in the thalamus after experimental TBI. (**A**), Representative photomicrographs of ipsi- and contralateral thalamic sections (LD) immunostained with MBP in the brains of control and CCI-injured rats at 3 months after experimental injury. EBA immunostaining was visualized with DAB (brown). The images demonstrate the distribution of EBA-immunoreactivity around microvessels in the thalamus. The open arrows indicate EBA-positive microvessels and yellow-filled arrows indicate EBA-immunonegative microvessels. Excerpts show zoomed area (×2.2) of the sections shown with rectangle marked (1) and (2) in contra- and ipsilateral thalamus, respectively and color deconvolution images demonstrating specific DAB visualization of EBA-positive and EBA-negative microvessels (brown). (**B**), Bar graph demonstrating the results of quantitative morphological analyses expressed as the relative density of the EBA-immunonegative microvessels as compared to contralateral hemisphere in control and CCI-injured rats at different time points after experimental TBI. *** *p* < 0.001, ** *p* < 0.01, * *p* < 0.05, two-way ANOVA (*n* = 4–9 rats per group). (**C**), Immunofluorescent co-localization of cleaved-caspase-3, EBA and DAPI in the thalamus following CCI. Representative photomicrographs of triple labeled fluorescent immunostaining in dorsal nucleus of thalamus of control and CCI-injured rat 3 months after experimental injury demonstrating localization of cleaved-caspase-3 (red) and EBA (green) immunoreactivities counterstained with DAPI (blue). Exposure in the immunofluorescence images was set to demonstrate morphological features of the cleaved caspase-3-immunopositive puncta that have intensity evidently higher than background and to demonstrate the absence of detectable cleaved-caspase-3 immunoreactivity in contralateral thalamus in the same experimental conditions. Excerpts (1) and (2) from the site ipsilateral to the injury demonstrate predominantly extracellular localization of cleaved-caspase-3-immunopositive puncta and diffuse matter (suggested by the lack of the co-localization of the most cleaved-caspase-3 immunoreactivity with DAPI or EBA) and limited association of cleaved-caspase-3 with EBA-labeled cells through direct or indirect interaction. Open white and filled yellow arrows in excerpt (1) demonstrate a limited possibly of intracellular or nuclear localization of cleaved-caspase-3 punctate and diffuse immunoreactivity, respectively (suggested by co-localization with DAPI). Open white arrows in the excerpt (2) indicate localization of punctate cleaved-caspase-3 immunoreactivities adjacent to blood-brain barrier (i.e., EBA-immunopositive cells). Red arrow indicates an example of co-localization of singular cleaved-caspase-3 punctum with EBA-labeled cell.

Our previous report suggested that upregulation of cleaved caspase-3 in the white matter of the corpus callosum is associated with microvascular damage at chronic time points after experimental TBI [39]. Cleaved-caspase-3 immunoreactivity in the selected thalamic regions was present as punctate staining, predominantly with extracellular localization, widely distributed in the affected areas and with perivascular localization. To quantitatively assess blood-brain barrier integrity in the thalamus, immunohistochemical experiments with EBA, a specific blood-brain barrier marker, were performed in the brain sections obtained from rats at different time points after CCI and control animals. EBA-immunopositivity was visualized with DAB stain (brown). EBA-immunopositive structures were predominantly distributed around lumina of microvessels with varied density in the ipsi- and contralateral thalamic regions. In the thalamus of control rats and in the contralateral hemisphere of CCI–injured animals, the vast majority of microvessels were immunopositive for EBA. In contrast, in the ipsilateral thalamus, the number of EBA-immunonegative microvessels was significantly increased in the areas associated with cleaved-caspase-3 upregulation as compared to the corresponding thalamic regions of the contralateral hemisphere. Figure 4A demonstrates differential distribution of EBA-positive and EBA-immunonegative microvessels in control and CCI-injured rats at 3 months after experimental TBI. Color deconvolution images demonstrate the different morphological features of the microvessels in the ipsi- and contralateral thalamic regions after CCI, such as decreased intensity or lack EBA-immunoreactivity surrounding microvessels in the ipsilateral regions. Quantitative morphological analyses revealed that significant increases in the numbers of EBA-immunonegative microvessels as compared to the contralateral side were observed starting at 1 week after CCI. These numbers remained increased up to 3 months following experimental TBI (Figure 4B). No statistically significant difference in total numbers of microvessels (i.e., EBA-immunonegative plus EBA-immunopositive) between ipsi- and contralateral thalamus was observed in any experimental group.

To further investigate possible involvement of cleaved-caspase-3 in chronic microvascular damage and blood-brain barrier breakdown in gray matter in the thalamus, we performed triple-immunofluorescence staining using EBA, a specific marker of the blood-brain barrier, counterstained with cleaved-caspase-3 and DAPI in control and CCI-injured rats at 3 months after experimental TBI or sham. The immunofluorescence-based experiments revealed prominent upregulation of cleaved-caspase-3 in the selected ipsilateral thalamic regions, primarily in dorsal areas adjacent to the hippocampal region and the fimbria of the hippocampus. No detectable cleaved-caspase-3 punctate immunoreactivity was observed in any of these regions in the contralateral hemisphere. Figure 4C demonstrates a typical morphological pattern of cleaved-caspase-3 at 3 months after experimental TBI. This figure shows that in the ipsilateral LD of thalamus in CCI-injured rats at 3 months after CCI, characteristically scattered immunopositivity for cleaved-caspase-3 manifested a predominantly extracellular localization (suggested by lack of co-localization of most cleaved-caspase-3 punctate staining with a nuclear DAPI counterstain). Figure 2 also demonstrates that in the ipsilateral thalamus, a substantial amount of cleaved-caspase-3-immunoreactivity was located within cellular structures, either as puncta surrounding DAPI-stained nuclei or as more diffuse immunopositive material located within DAPI-staining (shown in the excerpt marked (1) with white open and filled with yellow arrows, respectively). A fraction of DAPI-stained nuclei was negative for cleaved-caspase-3. Some fractions of the cleaved-caspase-3-immunopositive nuclei or cellular structures were located within EBA-positive cells, suggesting their direct interaction. These immunofluorescence images also demonstrate the limited presence of cleaved-caspase-3 puncta slightly overlying or touching the cellular structures immunopositive for the blood-brain barrier marker EBA [shown in the excerpt marked (2) with white open arrows]. However, the morphological patterns of cleaved-caspase-3 puncta immunoreactivity shown with white open arrows in the zoomed excerpt images provide evidence of a more indirect association with cleaved-caspase-3, probably within the cell membrane, rather than intracellular co-localization of cleaved-caspase-3 with EBA. Cleaved-caspase-3 immunopositive puncta predominantly showed an extracellular localization or co-localized with EBA-immunonegative cell types. Finally, the immunofluorescent images revealed the limited presence of cleaved-caspase-3 puncta co-localized with EBA [shown in the excerpt (2) with red arrow] suggesting direct involvement of caspase-3-mediated apoptosis with blood-brain barrier cells after TBI.

## 3. Discussion

The study investigated the role of activation of caspase-3-mediated apoptotic pathways in the chronic sequelae of TBI in rats using the well-established and characterized CCI model. These experiments focused on the immunohistochemical examination and analysis of the spatiotemporal profile of upregulation and distribution of cleaved-caspase-3, a specific effector of caspase pathway activation and its association with blood-brain barrier pathology and demyelination of neuronal processes in the gray matter of the thalamus. These results provide the first demonstration of significant chronic upregulation of cleaved caspase-3 in selected thalamic regions after CCI and the initial evidence that this upregulation of activated caspase-3 in the thalamus is associated with delayed degeneration of myelinated neurons and nerve fibers and loss of MBP integrity, thereby potentially affecting neuronal connectivity. Moreover, the results demonstrate for the first time previously unrecognized microvascular pathology associated with cleaved-caspase-3 in the thalamus involving microvascular reorganization with altered blood-brain barrier integrity.

### 3.1. Pathways and Mechanisms Involved in Chronic Pathologies in the Thalamus Following TBI

Our study provides experimental evidence of the involvement of activated caspase-3 in chronic consequences of TBI reflected in its upregulation in the gray matter of the thalamus for at least 3 months following TBI (the latest time point examined in the study). Chronic cleaved-caspase-3 increases were associated with neuronal demyelination in specific thalamic regions which are involved in important neurological functions. Disruption of thalamo-cortical connectivity is one of the recognized features of severe TBI [20,21,22,23]. Neuromodulatory interventions relevant to thalamic functions in the thalamus might promote late functional recovery [44]. Significantly, these thalamic regions are often impaired after TBI in humans, potentially causing or exacerbating common neurological complications associated with brain injuries, such as posttraumatic seizure and epilepsy, sleep disturbance and PTH [24,25,26,28]. Although the specific pathological pathways contributing to progressive neuronal degeneration after TBI have not been well defined, apoptotic cell death [33,35,45,46] and inflammation [47,48] have been commonly proposed as possible mechanisms underlying some chronic sequelae of brain trauma.

The mechanisms responsible for neurodegeneration in the brain following TBI involve several primary and secondary injury pathways, notably excitotoxic and apoptotic cell death involving calpain and caspase activation [49]. Clinical data provide evidence of the involvement of the caspase-3 in the acute and chronic consequences of TBI in different brain regions including cortex, hippocampus and thalamus [37]. However, previous preclinical studies using various TBI models including CCI have focused mostly on acute and sub-acute time points (up to four weeks). These studies documented upregulation of activated caspase-3 in the traumatized brain primarily in the cortex and hippocampus [50,51,52,53]. There is only limited preclinical evidence related to the subacute involvement of caspase-3-mediated apoptosis in the thalamus or other brain injury regions distal to the focal injury site [54,55,56]. To the best of our knowledge, there are no published data regarding chronic caspase-3-mediated pathways in these brain regions. Previous data obtained using the lateral fluid-percussion brain injury model in the rat have shown acute upregulation of cleaved caspase-3 and the presence of active caspase-3-immunopositive cells and apoptotic bodies in the thalamus staring at 3 and 7 days after experimental injury [54,55]. Another study using the lateral fluid-percussion TBI model reported an increase in the number of the apoptotic cells (i.e., positive for terminal deoxynucleotidyl transferase dUTP nick end labeling (TUNEL)) in the thalamus that peaked at two weeks and returned to control values at two months after injury [56]. In contrast, our study demonstrates that at the chronic time points, cleaved-caspase-3 upregulation was the most prominent in selected regions of ipsilateral thalamus, with predominantly extracellular localization, compared to other brain regions, including those directly impacted by experimental TBI. 

The mechanism triggering this abnormal expression of cleaved-caspase-3 is not completely understood. Published data suggest that white matter damage following TBI may play a critical role in the chronic progression of neuronal degeneration resulting in regional brain atrophy in distal areas that are not directly affected by primary injury [19], an observation consistent with our previous reports demonstrating chronic white matter degradation following focal cortical injury [38] and suggesting involvement of caspase-3-mediated apoptosis as a possible mechanism involved in this process [39]. The major impaired thalamic regions with the highest levels of cleaved-caspase-3 upregulation in our study include parts of the anterior and lateral groups of the dorsal thalamus, primarily LD and LP, respectively and parts of geniculate groups of both ventral and dorsal thalamus and, partially, in RT. To address potential association of cleaved-caspase-3 with neuronal pathologies, we compared the temporal profiles of cleaved-caspase-3 upregulation with histopathological findings of luxol fact blue and cresyl violet counterstaining, which are specific myelin and Nissl stains, respectively and MBP immunohistochemistry to assess anatomical alterations in myelin and cellular organization of the affected thalamic regions. The most prominent upregulation of cleaved caspase-3 in the thalamus occurred primarily in selected regions related to the sensory-motor and polymodal association cortices that have afferent connections with corresponding cortical regions prominently injured by CCI. These regions included primary and secondary motor areas, the primary somatosensory area, the retrosplenial area and the posterior and parietal association cortices [57,58,59]. The thalamic regions that are most susceptible to apoptosis in our chronic CCI model are also slightly different than those previously reported using the acute fluid percussion model [55], probably due to different injury modalities, injury severity and involvement of different cortical areas affected by experimental injury. At three months after CCI, the most obvious decreases in luxol fast blue staining intensity, indicative of myelin loss in the thalamus, were congruent with maximal increases in cleaved-caspase-3 immunoreactivity. These findings are consistent with decreases in MBP immunoreactivity associated with cell loss [60], suggesting a loss of myelinated neuronal fibers and viable cells. 

The spatiotemporal profile of luxol fast blue staining suggests that the myelin degradation in susceptible ipsilateral thalamic regions as compared to their contralateral counterparts begins as early as 24 h after CCI, whereas statistically significant persistent increases in cleaved-caspase-3 staining were observed one month following injury, thus suggesting that delayed apoptotic processes in the thalamus are triggered by disrupted connectivity with directly injured cortical regions. Experimental data also suggest that neuroinflammation involving activation of reactive microglia and astrocytes may play a role in delayed neurodegenerative processes in the thalamus after focal cortical injury in rodents [42,43,61,62]. Experimental studies performed using rodent CCI models have documented increases in the microglial markers in the most susceptible LD and LP of thalamus within the first two weeks after experimental injury, which at four weeks further expanded to the upper part of RT of thalamus [42,43,61,62]. Moreover, these experimental observations are consistent with clinical data indicating that, in severe TBI patients, persistent increases in microglial activation are present in selected thalamic regions remotely located from focal damage [63]. Importantly, although these thalamic regions were located in the ipsilateral hemisphere, they were not directly impacted by injury and were characterized by delayed cleaved-caspase-3 upregulation possibly associated with secondary apoptotic injury progression that could contribute to chronic neurological symptoms. 

Our data also suggest possible involvement of cleaved-caspase-3 by both direct and indirect mechanisms. The immunofluorescence data demonstrate that there is limited association of cleaved-caspase-3-positive puncta with EBA-immunopositive cells, despite the presence of an abundance of cleaved-caspase-3-positive puncta and diffuse immunoreactivity both extracellularly and intracellularly (i.e., co-localized with DAPI) in the surrounding areas (see excerpt (2) in Figure 2). This association is mostly reflected by adjacent direct localization of cleaved-caspase-3-positive puncta and/or DAPI-labeled structures co-localized with cleaved-caspase-3, suggesting that activation of caspase-3 could contribute to blood-brain barrier destruction through indirect mechanisms. In addition, the data further revealed limited co-localization cleaved-caspase-3-positive puncta with EBA, suggesting direct apoptotic mechanisms in the blood-brain barrier cells. Interestingly, a similar but less extensive pattern indicating association of cleaved-caspase-3-positive puncta staining with EBA-immunopositive cells was observed in corpus callosum at 3 months after CCI [39] suggesting the involvement of similar caspase-3-mediated mechanisms in white and gray matter regions not directly affected by injury. However, further studies are needed to establish the exact role of caspase-3 mediated apoptosis in delayed brain-barrier dysfunction following TBI. Our further investigation of the blood-brain barrier integrity in the thalamus revealed changes in microvascular organization reflected in significant decreases in density of EBA-immunopositive microvessels in the ipsilateral hemisphere, suggesting impaired blood-brain barrier function, which can further contribute to degenerative processes and the atrophy in the thalamus reported in clinical studies. The decreases in EBA-immunopositive microvessels were spatially and temporally associated with increases in cleved-caspase-3 immunoreactivity, suggesting involvement of caspase-3-mediated pathways. Investigation of the exact molecular and cellular mechanisms involved in abnormal microvascular reorganization is currently one focus of our ongoing studies. The possible mechanisms include loss of blood-brain barrier-positive cells due to cleaved-caspse-3-mediated apoptosis and/or abnormal angiogenesis involving formation of microvessels lacking a blood-brain barrier. 

### 3.2. Neurological Functions Associated with Thalamic Regions and Their Clinical Implications in Chronic Sequelae of TBI

Among the most impaired thalamic regions characterized by cleaved-caspase-3 upregulation and myelin loss (e.g., LP, LD, VGL, IGL) are functionally important structures playing a critical role in maintaining normal neurological function. For example, LP LD of thalamus are involved in innervation of thalamo-cortical and cortico-striatal-thalamic networks responsible for visual processing [58,64,65], spatial processing, orientation, directed attention, spatial learning and memory [66,67,68] and contextual fear conditioning [69]. Additionally, VLG and IGL of thalamus are responsible for color discrimination, distinguishing among light intensities and some aspects of the regulation of circadian rhythms [70]. Our study also demonstrates a significant upregulation of cleaved caspase-3 in DLG, suggesting the existence of delayed apoptotic pathways starting at one month after brain injury that could exacerbate neurodegeneration in this region and underline chronic pathological alterations in the mammalian visual system following TBI [71,72]. The thalamo-cortical networks, including selected cortical areas that are affected by CCI (e.g., somatomotor, somatosensory and polymodal cortical areas), are also involved in the rodent nociceptive system [73]. Importantly, inflammation and altered neuroplasticity in the cortical barrel circuit and thalamus following diffuse fluid percussion injury were associated with hypersensitivity to whisker stimulation and allodynia [74,75,76], processes associated with central pain mechanisms. 

Further preclinical data suggest that chronic anatomical and immunohistochemical alterations in the thalamus following TBI might be associated with neurological and behavioral complications in humans including sleep disturbances [62,77] and chronic headaches [75,76,78], both common complications of brain trauma which can affect the clinical outcomes and quality of life of TBI survivors. Pathological functional, anatomical and metabolic alterations in the aforementioned thalamic regions affected by CCI in our chronic experimental model have been also described in other animal models of brain injuries, neurological disorders and neurotoxicity resulting in impaired cognitive and behavioral outcomes [79,80,81,82]. Interestingly, increased caspase-3 expression and a higher number of degenerating neurons was observed in the LD of thalamus in a genetic rat epilepsy model with associated cognitive deficits [81] and in the developing rodent brain after ethanol exposure [83]. 

Clinical implications, study limitations and future directions.

The apparent homology between rat and human thalamic projections involving thalamic regions chronically affected by CCI in our study have been described for the LP of thalamus [84,85]. In humans, thalamo-cortical network activity is important for regulation of sleep-wake states and dysfunction of this network is linked to a number of neuropsychiatric conditions collectively known as thalamo-cortical dysrhythmia syndrome [86,87]. On the other hand, sleep disruption has a negative effect on the neural remodeling necessary for recovery from TBI and may prolong rehabilitation by impeding these processes [88,89]. 

Taken together with the data outlined above, the present observations suggest that delayed cleaved-caspase-3 upregulation and chronic apoptosis in the thalamus might be important common mechanisms underlying neurological and behavioral deficits that are often associated with chronic sequelae of TBI. In addition, caspase-3 activation could be one of the early secondary mechanisms triggering development of neurological and neurodegenerative diseases for which TBI is a risk factor. Considering the heterogeneity of human TBI, it is possible that the primary focal impact to the specific cortical zone will initiate delayed apoptotic processes in corresponding thalamic region(s) that will predispose to delayed chronic neurological impairments. Intriguingly, based on these assumptions, cortical injury location and phenotype evaluated by brain imaging could provide important information regarding prognosis of chronic clinical outcomes. The delayed nature of caspase-3-mediated apoptotic processes in the affected thalamic regions suggests that this pathway is a promising target for the effective treatment and prophylaxis of chronic neurological and behavioral complications following brain trauma. 

Some limitations of this study include the observation that the data were obtained using immuno- and histochemical methods and did not involve assays of caspase-3 activation and quantification of its proteolytic products in tissue, an approach that would provide more rigorous biochemical clarification of the temporal immunohistochemical changes associated with cleaved-caspase-3 activity. Thus, future studies will be focused on investigation of the specific cell type involvement that led to accumulation of extracellular cleaved-caspase-3 puncta in thalamus. Additional studies are warranted to determine spatiotemporal profiles of some important caspase-3-mediated protein cleavage products reported in patients with brain injuries such as caspase-3-cleaved cytokeratin-18 (CCCK-18) [90] and 120- and 150-kDa αII spectrin breakdown products (SBDP120 and SBDP150, respectively) [91,92,93] (see also review by Glushakova and colleagues [37]). In addition, our future research will be focused on further studies of pathological pathways associated with accumulation of caspase-3-cleaved tau, a specific cleavage product of caspase-3-mediated proteolysis, which might be involved in development of chronic neuropathology following TBI and serve as a novel biomarker of chronic TBI associated with neurodegeneration [39]. These studies will provide further insights into the potential role of caspase-3-mediated pathways in microvascular, neuronal and behavioral pathologies associated with chronic TBI, thereby extending potential clinical applications for utilization of these pathways for diagnostic applications and as targets for treatment of brain injuries. 

## 4. Materials and Methods

### 4.1. Animals

Adult male Sprague-Dawley rats weighing 230 to 300 g (Harlan, Inc. Indianapolis, IN, USA) were maintained in a specialized temperature—(20–24 °C) and humidity—(30–70%) controlled facility on a twelve-hour light/dark cycle with access to a normal laboratory diet and potable water ad libitum. They were acclimated to this environment for at least one week before being used in the study. All experimental procedures were approved by the University of Florida IACUC (201005012, 21 October 2010). In all animals, pre- and post-surgery pain management was maintained to ensure compliance with guidelines set forth by the University of Florida IACUC.

### 4.2. Controlled Cortical Impact (CCI)

Mild-to-moderate experimental TBI was induced using Benchmark™ Stereotaxic Impactor (MyNeurolab, St. Louis, MO, USA) as previously described elsewhere [38,39]. Briefly, all surgical procedures were performed under isoflurane anesthesia (4% induction and 2.5% maintenance in oxygen). Unilateral CCI was produced by a single impact using a cylindrical impactor with tip diameter of 4 mm. The CCI parameters were as follows: velocity 3.5 m/s, compression distance 2.5 mm and compression time 200 ms. Sham animals underwent anesthesia and all surgical procedures including craniotomy but not cortical impact. After surgery, rats were allowed to completely recover from anesthesia in a temperature-controlled recovery chamber and transferred to a housing facility where they were monitored for up to 3 months following CCI.

### 4.3. Immunohistochemical Analysis

The coronal brain sections used in this study were selected from the brain segment located within the midline of the circular craniotomy, which corresponded to the impact zone representing reproducible injury profiles in all major brain regions affected by focal CCI injury, including the cortical core lesion and penumbra as well as regions that are not directly impacted, such as the thalamus [38,39,94,95]. Immunohistochemistry on paraffin-embedded 4-µm coronal sections from stereotaxically corresponding thalamic regions from a brain segment positioned within the midline plane of impact site (between −3.8 mm and −2.8 mm from bregma) was performed as detailed in our previous study [38,39,96] with minor modifications. In this study, the following primary antibodies with dilutions 1:500 or 1:1000 depending on applications were used: (1) rabbit polyclonal anti-cleaved caspase-3 (Asp175) (Cat# 9661; Cell Signaling Technology, Danvers, MA, USA); (2) mouse monoclonal anti-rat blood-brain barrier [endothelial barrier antigen (EBA)] (Cat #SMI-71R, BioLegend, San Diego, CA, USA); and rabbit anti-myelin basic protein (MBP) (Cat# AB5864, EMD Millipore, Billerica, MA, USA). According to manufacturer’s data, cleaved-caspase-3 (Asp175) antibody detects endogenous levels of the large fragment of activated caspase-3 (17/19 kDa) resulting from cleavage adjacent to Asp175 and this antibody does not recognize full length caspase-3 (35 kDa) or other cleaved caspases. For the quantitative immunohistochemical experiments we used biotinylated secondary antibodies and avidin-HRP conjugate (LSAB+ kit, Cat #K0679, Dako, Carpinteria, CA, USA). Immunostaining was visualized with 3,3′-diaminobenzidine (DAB) for brown color development and sections were counterstained with hematoxylin (Dako, Carpinteria, CA, USA). To visualize myelin and brain tissue pathology, the sections were stained with luxol fast blue and counterstained with cresyl violet using a commercially available kit (NovaUltra™ Luxol Fast Blue Stain Kit, IHC WORLD, LLC, Woodstock, MD, USA). For the immunofluorescence triple-labeling experiments, the following species-specific secondary antibodies conjugated with Alexa Fluor dyes (all from Thermo Fisher Scientific, Waltham, MA, USA) were used: (1) donkey anti-rabbit IgG/Alexa Fluor^®^ 555 (Cat #A-31572), (2) chicken anti-mouse IgG/Alexa Fluor^®^ 488 (Cat# A-21200) and chicken anti-goat IgG/Alexa Fluor^®^ 488 (Cat# A-21467) and 4′,6-diamidino-2-phenylindole (DAPI) was used for visualization of nuclei.

The slides were scanned and examined using Aperio ScanScope GL (Aperio Technologies, Vista, CA, USA) system using ×20 objective. Quantitative immunohistochemical analyses in scanned slide images were performed using the Positive Pixel Count algorithm included in the ImageScope software Version 12.3.2.813 (Aperio Technologies) in the areas corresponding to specific thalamic regions of the ipsilateral hemisphere with increased immunopositivity (notably at chronic time points). The same thalamic regions were identified and annotated for the morphological and quantitative analyses in in all animals including controls and were expressed as positive pixel numbers per mm^2^. Parameters for this algorithm were set to detect only positive pixels with significantly higher intensities than background level and accuracy of data was confirmed visually using markup images. Visualization of the specific stains for morphological analyses of scanned slide images was performed using the Color Deconvolution algorithm included in the ImageScope software (Aperio Technologies). Quantitative morphological analyses of microvessels were performed using ImageJ software (National Institutes of Health, Bethesda, MD, USA). Triple immunofluorescence-labeled sections were examined using the Olympus IX70 Inverted Fluorescent Microscope equipped with a ×60 objective and QImaging Retiga 4000R Monochrome Camera with RGB-HM-5 Color Filter and analyzed using QImaging QCapture Pro 6.0 image analysis software.

### 4.4. Statistical Analysis

For statistical analysis of specific immunostaining, one-way ANOVA followed by Newman-Keuls multiple comparison tests or two-way ANOVA followed by Bonferroni post-test (Prism 5, Graphpad, La Jolla, CA, USA) were used. Data were reported as mean ± SEM. *p* Values less than 0.05 were considered significant. The specific numbers of rats are shown in all corresponding figures and results of quantitative analyses.

## 5. Conclusions

This study provides new insights into potential mechanisms involving chronic activation of caspase-3 associated with disrupted cortico-thalamic and thalamo-cortical connectivity after experimental TBI. Our data provide the first evidence that activation of caspase-3 is directly associated with myelin pathology, blood-brain barrier alterations and thalamic degeneration. Caspase-3 activation could further contribute to delayed axonal damage and induce apoptosis across thalamo-cortical networks. Moreover, upregulation of activated caspase-3 and delayed degeneration of myelinated neurons and nerve fibers in the thalamus, along with microvascular reorganization with impairment of blood-brain barrier integrity, might be representative of reciprocal pathological processes affecting neuronal connectivity and brain function during chronic stages of TBI, thereby implicating the caspase-3-mediated pathway as a promising target for development of novel diagnostic and therapeutic strategies for chronic monitoring and management of TBI patients. 

## Abbreviations

TBI	Traumatic brain injury
CCI	Controlled cortical impact
MBP	Myelin basic protein
EBA	Endothelial barrier antigen
PTH	Posttraumatic headaches
CNS	Central nervous system
CSF	Cerebrospinal fluid
DAPI	4′,6-diamidino-2-phenylindole
DAB	3,3′-diaminobenzidine
LP	Lateral posterior thalamic nucleus
LD	Laterodorsal thalamic nucleus
DLG	Dorsal lateral geniculate nucleus
VLG	Ventral lateral geniculate nucleus
RT	Reticular thalamic nucleus
IGL	Intergeniculate leaf
IMA	Intramedullary thalamic area
MD	Mediodorsal thalamic nucleus
Po	Posterior thalamic nuclear group
VPL	Ventral posterolateral thalamic nucleus
VPM	Ventral posteromedial thalamic nucleus
CL	Centrolateral thalamic nucleus
OPC	Oval paracentral thalamic nucleus
TUNEL	Terminal deoxynucleotidyl transferase dUTP nick end labeling
CCCK-18	Caspase-3-cleaved cytokeratin-18
SBDP120	120 and 150 kDa αII spectrin breakdown product
SBDP150	150 kDa αII spectrin breakdown product
